# Supplementary material for: The Discovery of New Inhibitors of Insulin-Regulated Aminopeptidase by a High-Throughput Screening of 400,000 Drug-like Compounds
Source: Int J Mol Sci. 2024 Apr 6;25(7):4084. doi: 10.3390/ijms25074084 (PMC11012289; doi:10.3390/ijms25074084)
Supplement: Supplementary file 1 [file ijms-25-04084-s001.zip › ijms-2920019-supplementary.pdf]

The Discovery of New Inhibitors of Insulin Regulated Amino Peptidase by A High-Throughput Screening of 400 000 Drug-like Compounds

Johan Gising[1,*], Saman Honarnejad[2], Maaike Bras[2], Gemma L. Baillie[3], Stuart P. McElroy[3], Philip S. Jones[3], Angus Morrison[3], Julia Beveridge[1], Mathias Hallberg[4], Mats Larhed[1]

[1] The Beijer Laboratory, Science for Life Laboratory, Department of Medicinal Chemistry, Uppsala University, BMC, P.O. Box 574, SE-751 23 Uppsala, Sweden
[2] Pivot Park Screening Centre, Kloosterstraat 9, 5349 AB, Oss, The Netherlands
[3] BioAscent Discovery Ltd, Bo 'Ness Road, Newhouse, Lanarkshire ML1 5UH, United Kingdom
[4] The Beijer Laboratory, Department of Pharmaceutical Biosciences, Neuropharmacology and Addiction Research, BMC, Uppsala University, P.O. Box 591, SE-751 24 Uppsala, Sweden
[*]Corresponding author, johan.gising@angstrom.uu.se

Supplementary materials

Table of contents:

Equations S1 ………...2
Fig. S1. Reference compound structures: **IVDE-77**, **HA08** and **I**……………………………………………………...2
Fig. S2. Assay principle of the IRAP assays…………………………………………………………………...........2
Fig. S3. Km determination for the IRAP Fluorescence assay in 1536-well format..……………………..…..3
Fig. S4. Km determination for the IRAP Absorbance assay in 1536-well format…………………………....3
Fig. S5. Fluorescence IRAP assay with black non-binding plates and 0.05% BSA added to the assay buffer in 1536-well format……………………………………………………………………………………….....4
Fig. S6. Absorbance IRAP assay with black non-binding plates and 0.05% BSA added to the assay buffer in 1536-well format………………………………………………………………………………………….....4
Fig. S7. Absorbance IRAP assay with black non-binding plates and 0.05% BSA added to the assay buffer in 1536-well format……………………………………………………………………………………….....5
Fig. S8. Fluorescence IRAP assay reagent stability……………………………………………………………..…..5
Fig. S9. Reference compounds in Fluorescence IRAP assay with different end volumes in 1536-well format………......6
Fig. S10. Fluorescence IRAP assay with automated dispensing; S/B ratio, Z'-values and %CV……...…7
Fig. S11. Robustness set tested on duplicate plates for the Fluorescence IRAP assay…………………......7
Fig. S12. Thermal Shift assay dose response results of **HA08** and **I**……………………………………...…8
Fig. S13. S/B (blue bars) and Z' values (red dots) for the primary and orthogonal IRAP assay………...9
Fig. S14. Structures of remaining QHL compounds……………………………………………………….....10
Table S1. Summarized screening results and compound properties.……………………………………....11
Table S2. Compound ELF screening history………………………………………………………………………...12
References………...12

Equations S1

% Effect $= \frac{(\mu p - \mu n) - (\mu p - X)}{(\mu p - \mu n)} * 100$; measured value (X), mean (μ), positive (p) and negative (n) control

S/B signal/background ratio

Z' $= 1 - \frac{3(\sigma p + \sigma n)}{|\mu p - \mu n|}$; standard deviation (σ), mean (μ), positive (p) and negative (n) control

Z-score $= \frac{X - \mu n}{\sigma n}$; measured value (X), standard deviation (σ), mean (μ), negative (n) control

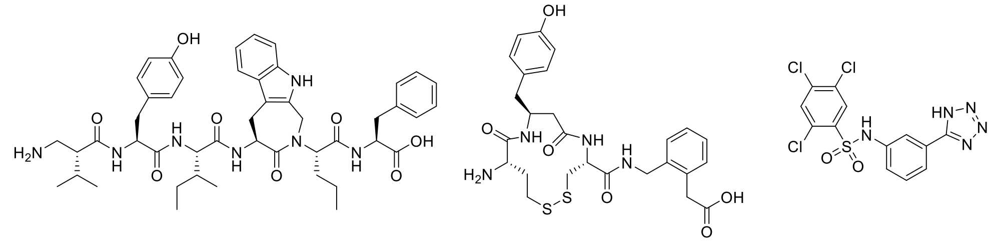

IVDE77 HA08 I

Figure S1. Reference compound structures. IVDE77[1], **HA08**[2], **I**[3].

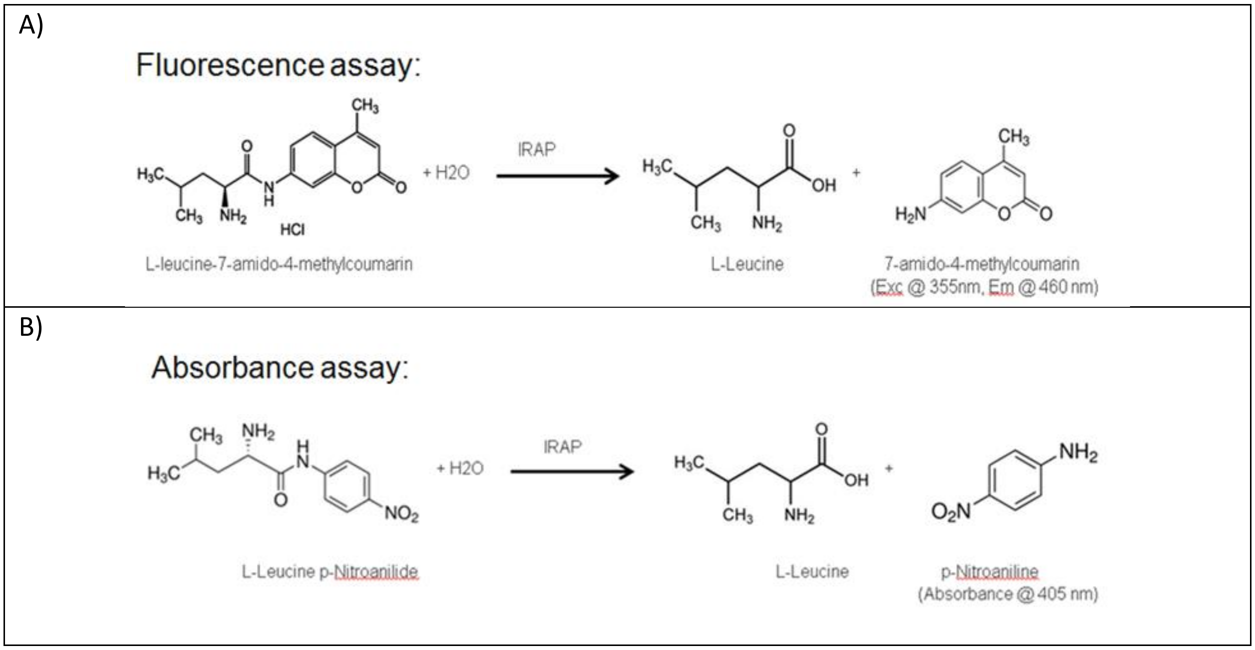

Figure S2. Assay principle of the IRAP assays. The substrates (A) L-Leucine-7-amido-4-methylcoumarin (Fluorescence) or (B) L-Leucine p-Nitroanilide (Absorbance) can be cleaved by the IRAP protein resulting in the amino acid L-Leucine and a measurable group. For the fluorescence assay 7-amido-4-methylcoumarin can be excited at 355 nM after which emission can be measured at 460 nm. For the absorbance assay the absorbance of p-Nitroaniline can be measured at 405 nM. When a compound inhibits the IRAP protein, the cleavage of the substrates will not take place and no signal will be measured.

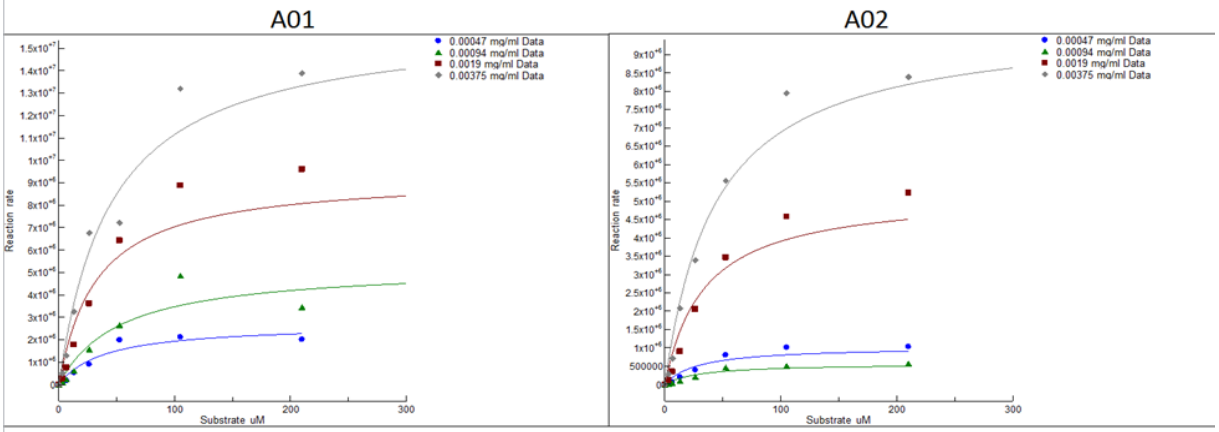

	0.00375 mg/ml	0.00188 mg/ml	0.00094 mg/ml	0.00047 mg/ml	
A01	45.1 µM	32.5 µM	52.5 µM	39.4 µM	
A02	43.6 µM	33.8 µM	30.3 µM	28.7 µM	

Figure S3. Km determination for the IRAP Fluorescence assay in 1536-well format. Km calculation at 4 protein concentrations (0.00375, 0.00188, 0.00094 and 0.00047 mg/mL) using Xlfit version 5.4.0.8.

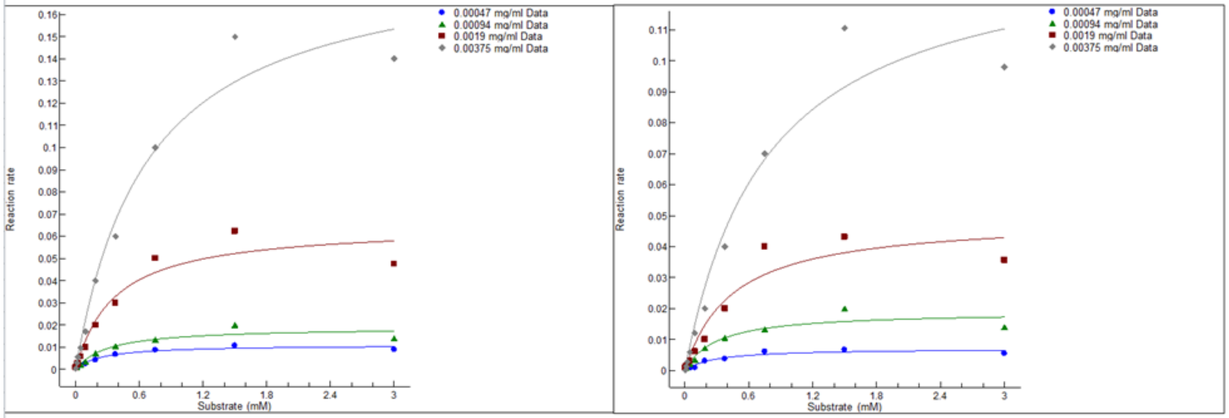

	0.00375 mg/ml	0.00188 mg/ml	0.00094 mg/ml	0.00047 mg/ml	
A01	0.67 mM	0.36 mM	0.30 mM	0.24 mM	
A02	0.78 mM	0.44 mM	0.30 mM	0.27 mM	

Figure S4. Km determination for the IRAP Absorbance assay in 1536-well format. Km calculation at 4 protein concentrations (0.00375, 0.00188, 0.00094 and 0.00047 mg/ml) using Xlfit version 5.4.0.8.

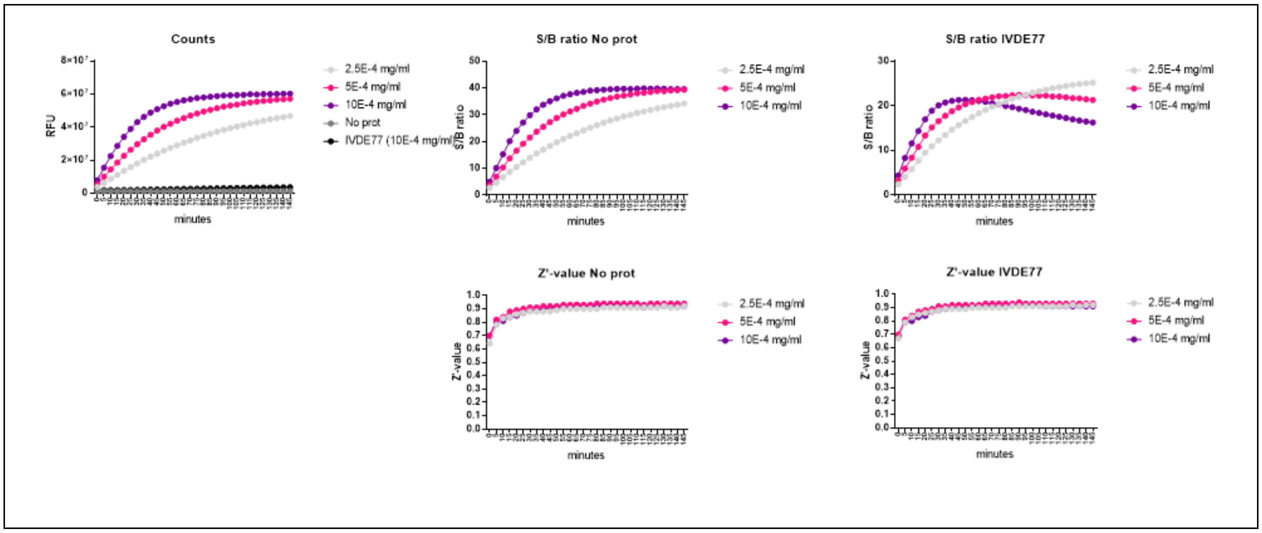

Figure S5. Fluorescence IRAP assay with black non-binding plates and 0.05% BSA added to the assay buffer in 1536-well format. Counts, S/B ratio and Z'-value. In 1536-well 2 µL buffer with DMSO or compound (**IVDE77**, **HA08** or **I**), 2 µL IRAP (f.a.c. 10E-4, 5E-4 or 2.5E-4 mg/ml) and 4 µL L-Leucine-7-amido-4-methylcoumarin (f.a.c. 37.5 µM) were manually dispensed in a 1536-well black non-binding assay plate (Corning, #3728). For the MAX effect either no protein (2 µL assay buffer was added instead of IRAP) or 1 µM **IVDE77** was included. Plates were measured on the Envision every 5 minutes for 2.5 hours.

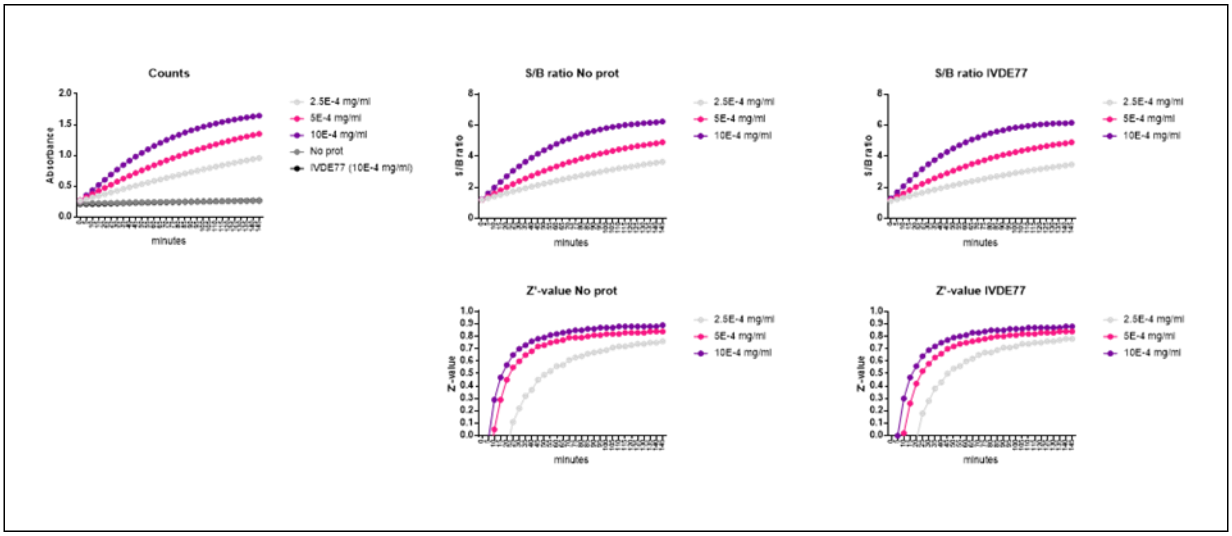

Figure S6. Absorbance IRAP assay with black non-binding plates and 0.05% BSA added to the assay buffer in 1536-well format. Counts, S/B ratio and Z'-value at 0.4 mM substrate. In 1536-well 2 µL buffer with DMSO or compound (**IVDE77**, **HA08** or **I**), 2 µL IRAP (f.a.c. 10E-4, 5E-4 or 2.5E-4 mg/ml) and 4 µL L-Leucine p-Nitroanilide (f.a.c. 0.4 mM) were manually dispensed in a 1536-well non-binding black view assay plate (Corning, #3895). For the MAX effect either no protein (2 µL assay buffer was added instead of IRAP) or 1 µM **IVDE77** was included. Plates were measured on the Envision every 5 minutes for 2.5 hours.

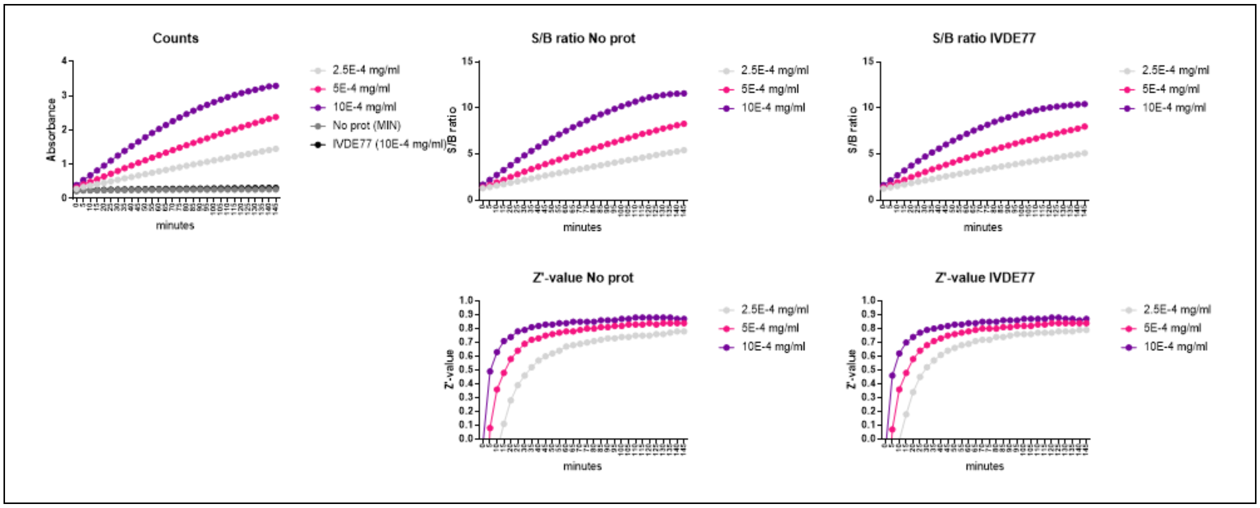

Figure S7. Absorbance IRAP assay with black non-binding plates and 0.05% BSA added to the assay buffer in 1536-well format. Counts, S/B ratio and Z'-value at 1 mM substrate. In 1536-well 2 µL buffer with DMSO or compound (**IVDE77**, **HA08** or **I**), 2 µL IRAP (f.a.c. 10E-4, 5E-4 or 2.5E-4 mg/mL) and 4 µL L-Leucine p-Nitroanilide (f.a.c. 1 mM) were manually dispensed in a 1536-well non-binding black view assay plate (Corning, #3895). For the MAX effect either no protein (2 µL assay buffer was added instead of IRAP) or 1 µM **IVDE77** was included. Plates were measured on the Envision every 5 minutes for 2.5 hours.

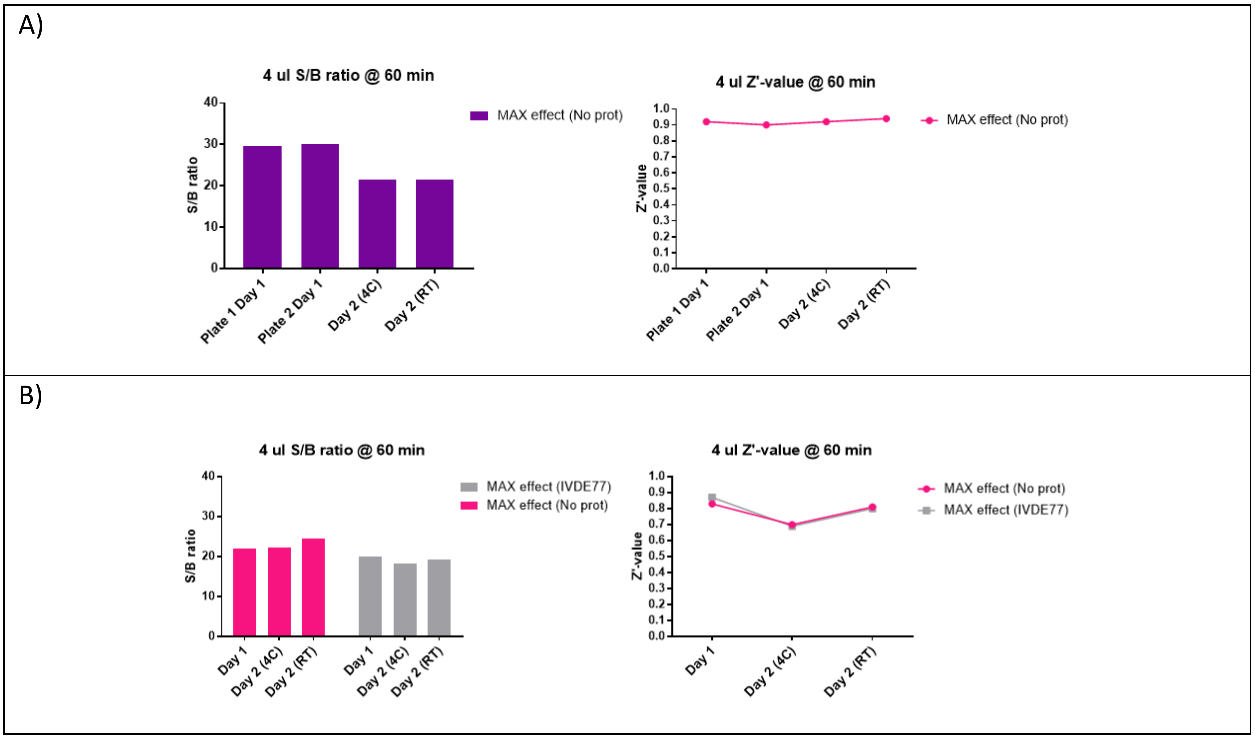

Figure S8. Fluorescence IRAP assay reagent stability. A) With the ECHO 10 nL DMSO was transferred to a black non-binding assay plate (Corning #3728). 2 µL IRAP (f.a.c. 2.5E-4 mg/mL) was added using the Certus and 2 µL L-Leucine-7-amido-4-methylcoumarin (f.a.c. 37.5 µM) was added using the FLEXdrop. In the MAX effect wells 2 µL assay buffer was added instead of IRAP using the FLEXdrop. Plates were measured on Envision after 1 hour at RT in the dark. B) In 1536-well 1 µL buffer with DMSO/**IVDE77** (f.a.c. 0.25%/1 µM), 1 µL IRAP (f.a.c. 2.5E-4 mg/mL) and 2 µL L-Leucine-7-amido-4-methylcoumarin (f.a.c. 37.5 µM) were manually dispensed in a 1536-well black non-binding assay plates (Corning, #3728). Plates were measured on the Envision every 10 minutes for 140 minutes (only 60 minute time point shown).

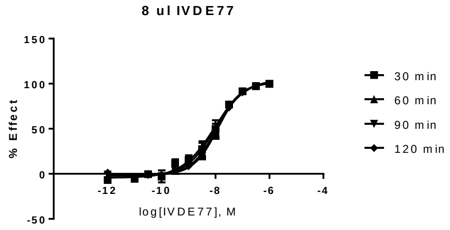

	30 min	60 min	90 min	120 min
Best-fit values				
Bottom	-4.409	-2.389	-1.693	-1.433
Top	105.3	103	101.9	101.5
LogEC50	-8.049	-8.022	-7.966	-7.92
HillSlope	0.7257	0.8414	0.9332	0.969
EC50	8.938e-009	9.499e-009	1.08e-008	1.202e-008

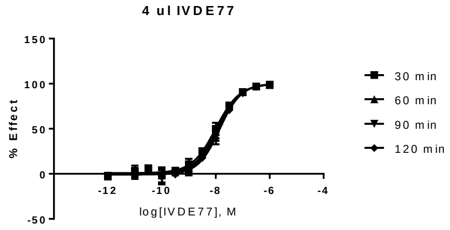

	30 min	60 min	90 min	120 min
Best-fit values				
Bottom	0.8847	0.05632	-0.7813	-0.9048
Top	99.71	99.88	100.2	100
LogEC50	-7.999	-7.949	-7.901	-7.852
HillSlope	0.9943	1.032	1.039	1.091
EC50	1.002e-008	1.125e-008	1.257e-008	1.404e-008

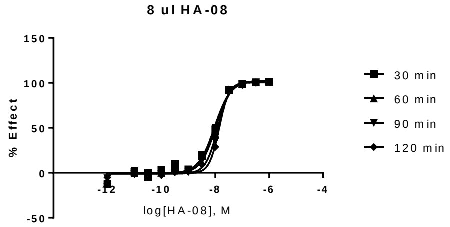

	30 min	60 min	90 min	120 min
Best-fit values				
Bottom	-0.6828	-0.9878	-1.142	-0.6506
Top	102.4	102.3	101.4	100.4
LogEC50	-8.022	-7.981	-7.905	-7.855
HillSlope	1.452	1.533	1.931	2.376
EC50	9.507e-009	1.045e-008	1.246e-008	1.397e-008

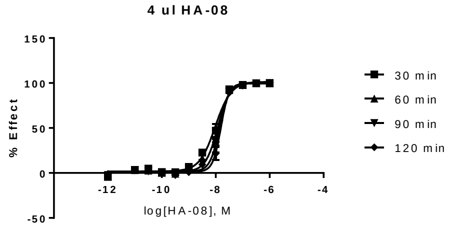

	30 min	60 min	90 min	120 min
Best-fit values				
Bottom	1.825	1.475	1.803	1.41
Top	101.4	100.8	99.84	99.47
LogEC50	-8.016	-7.921	-7.859	-7.808
HillSlope	1.485	1.899	2.48	2.91
EC50	9.648e-009	1.199e-008	1.383e-008	1.557e-008

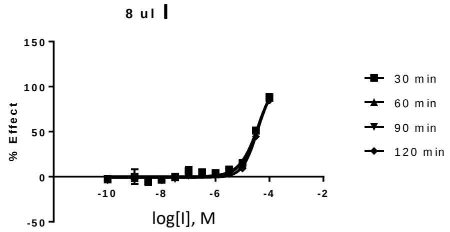

	30 min	60 min	90 min	120 min
Best-fit values				
Bottom	0.5937	-0.4046	-1.096	-1.198
Top	118.5	109.4	102.7	103.1
LogEC50	-4.385	-4.449	-4.469	-4.427
HillSlope	1.213	1.355	1.543	1.567
EC50	4.118e-005	3.557e-005	3.397e-005	3.74e-005

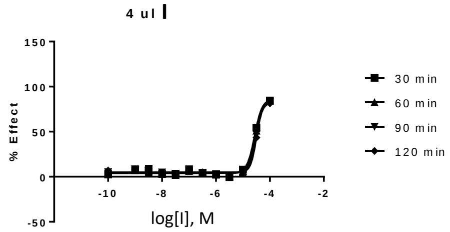

	30 min	60 min	90 min	120 min
Best-fit values				
Bottom	4.201	3.997	4.586	4.844
Top	86	85.3	83.47	82.11
LogEC50	-4.565	-4.541	-4.517	-4.5
HillSlope	3.12	3.375	3.729	4.01
EC50	2.723e-005	2.879e-005	3.042e-005	3.165e-005

Figure S9. Reference compounds in Fluorescence IRAP assay with different end volumes in 1536-well format. S/B ratio and Z'-values; Reference compound DRC's. In 1536-well 1 or 2 µL buffer with compound (**IVDE77** and **HA08**: 1 µM to 10 pM, **I**: 100µM – 1 nM (All compounds 12 points, √10 dilution; last point DMSO only)), 1 or 2 µL IRAP (f.a.c. 2.5E-4 mg/ml) and 2 or 4 µL L-Leucine-7-amido-4-methylcoumarin (f.a.c. 37.5 µM) were manually dispensed in a 1536-well black non-binding assay plate (Corning, #3728). Plates were measured on the Envision every 10 minutes for 140 minutes (data shown up to 2 hours). %Effect was calculated using no protein as MAX effect (assay buffer was added instead of IRAP).

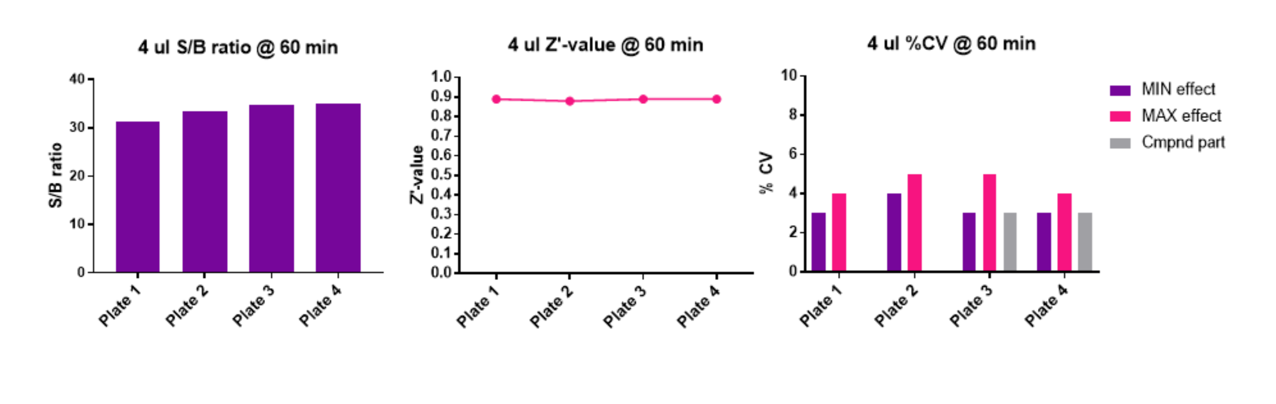

Figure S10. Fluorescence IRAP assay with automated dispensing; S/B ratio, Z'-values and %CV. With the ECHO 10 nL compound/DMSO was transferred to a black non-binding assay plate (Corning #3728). 2 µL IRAP (f.a.c. 2.5E-4 mg/mL) was added using the Certus and 2 µL L-Leucine-7-amido-4-methylcoumarin (f.a.c. 37.5 µM) was added using the FLEXdrop. In the MAX effect wells 2 µL assay buffer was added instead of IRAP using the FLEXdrop. Plates were measured on Envision after 1 hour at RT in the dark.

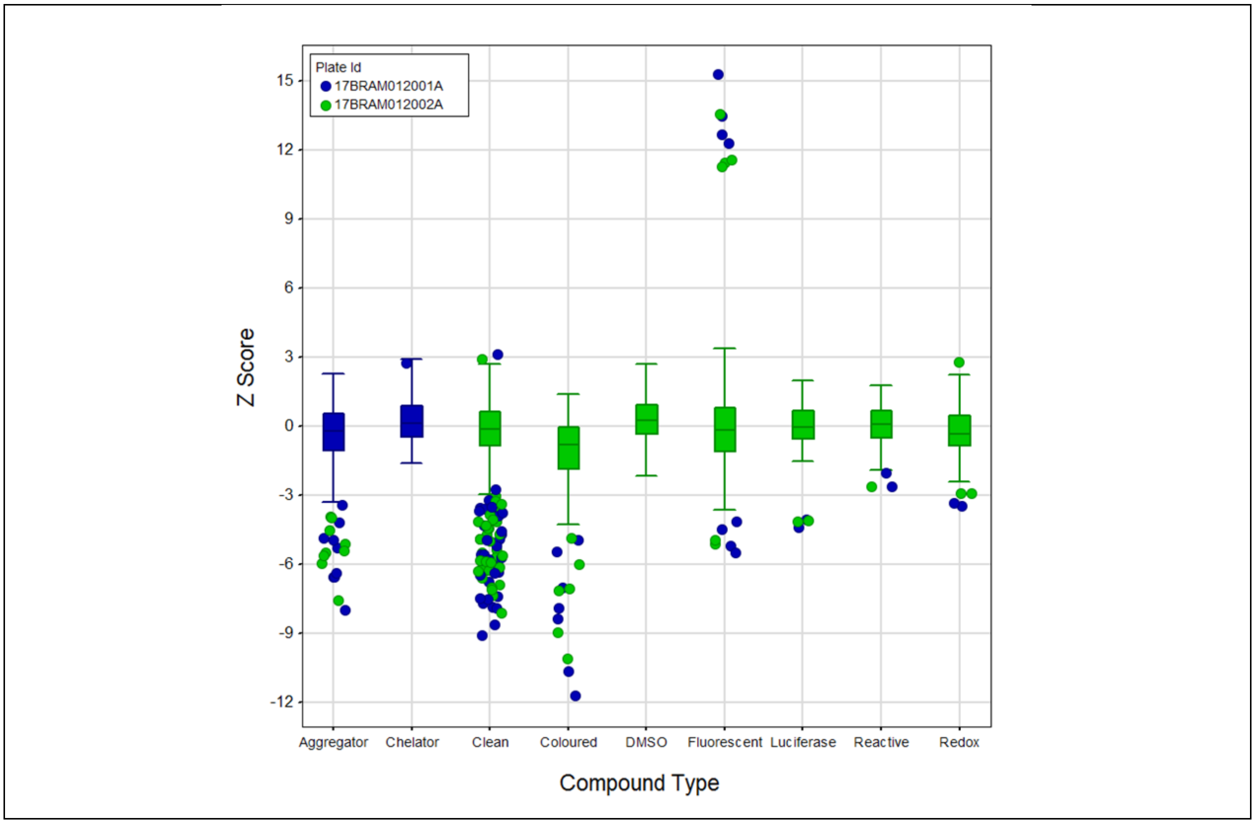

Figure S11. Robustness set tested on duplicate plates for the Fluorescence IRAP assay. Z-score for the robustness set compounds. Individual dots indicate the individual well values (compounds were tested in quadruplicate per plate), the color indicates the plate number (blue: 17BRAM012001A, green: 17BRAM012002A). With the ECHO 10 nL compound/DMSO was transferred to a black non-binding assay plate (Corning #3728). 2 µL IRAP (f.a.c. 2.5E-4 mg/ml) was added using the Certus and 2 µL L-Leucine-7-amido-4-methylcoumarin (f.a.c. 37.5 µM) was added using the FLEXdrop. Z-score was calculated based on the MIN effect wells (10 nL DMSO + 2µL IRAP + 2µL Substrate). Plates were measured on Envision after 1 hour at RT in the dark. Graphs generated using Dotmatics Vortex software.

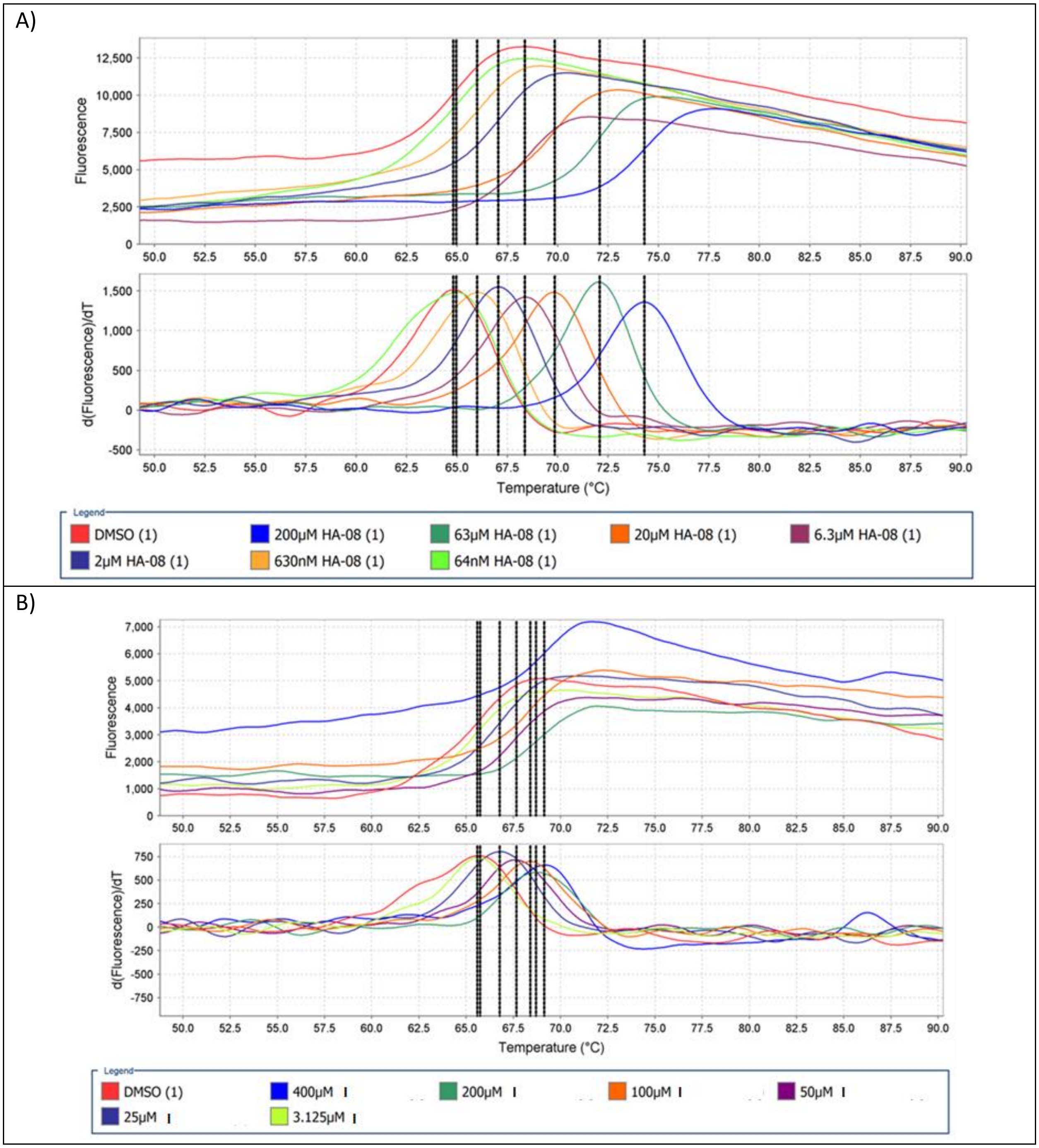

Figure S12. Thermal Shift assay dose response results of HA08 and I. Denaturation temperature of IRAP protein with DMSO and various concentrations of A) **HA08** and B) **I**.

A)

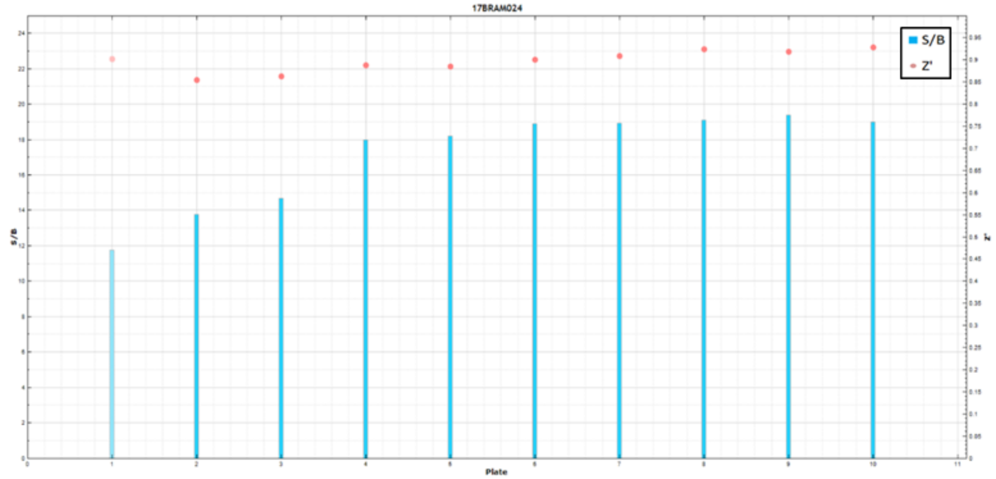

Figure S13A. S/B (blue bars) and Z' values (red dots) for the primary IRAP assay. With the ECHO 10 nL compound (f.a.c. 10 µM) was transferred to a black non-binding assay plate (Corning #3728) after which the plates were manually sealed using adhesive seals. The next day 2 µL IRAP (f.a.c. 2.5E-4 mg/mL) was added using the Certus and 2 µL L-Leucine-7-amido-4-methylcoumarin (f.a.c. 37.5 µM) was added using the FLEXdrop. In the MAX effect wells 2 µL assay buffer was added instead of IRAP using the FLEXdrop. Plates were measured on the Envision after 1 hour at RT in the dark.

B)

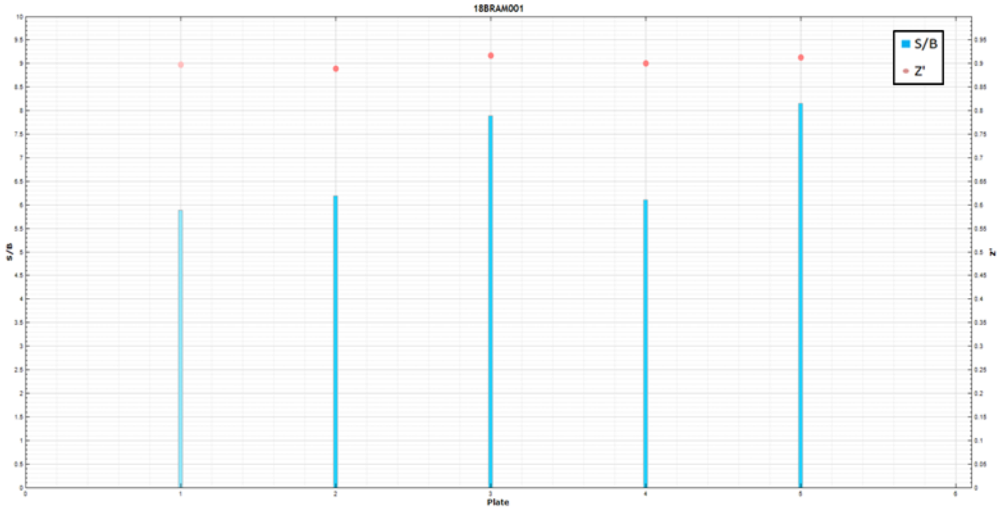

Figure S13B. S/B (blue bars) and Z' values (red dots) for the orthogonal IRAP absorbance assay. With the ECHO 20 nL compound was transferred to a black non-binding view assay plate (Corning #3895) after which the plates were manually sealed using adhesive seals. The next day 4 µL IRAP (f.a.c. 2.5E-4 mg/mL) was added using the Certus and 4 µL L-Leucine p-Nitroanilide (f.a.c. 1 mM) was added using the FLEXdrop. In the MAX effect wells 4 µL assay buffer was added instead of IRAP using the FLEXdrop. Plates were measured on the Envision after 2 hours at RT in the dark.

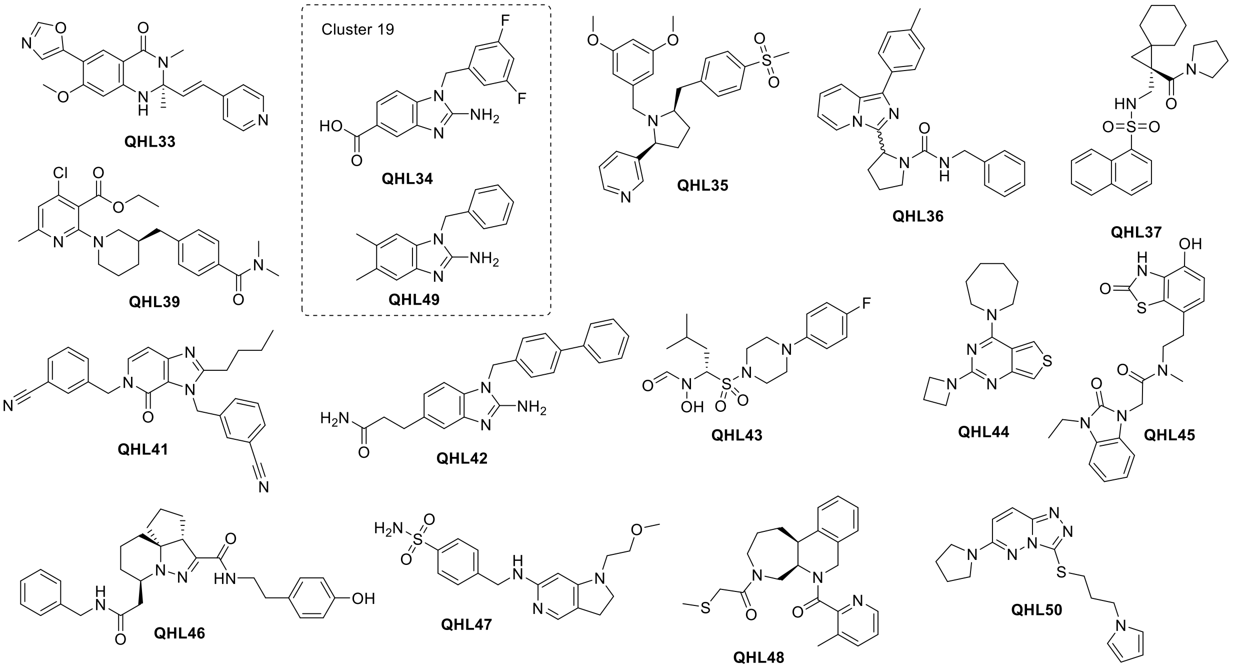

Figure S14. Structures of remaining QHL compounds.

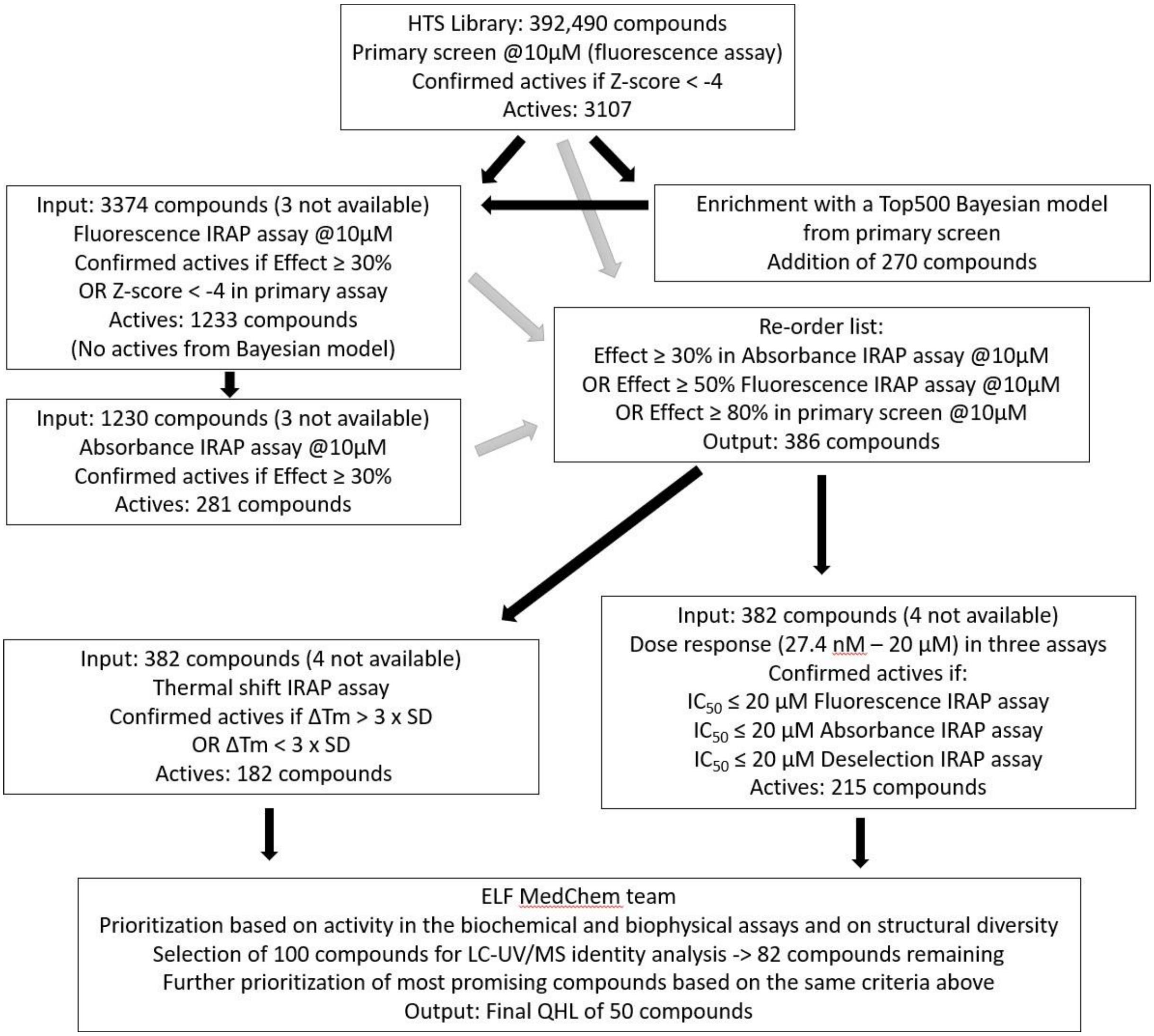

Figure S15. Screening funnel with decision points.

Table S1. Summarized screening results and compound properties.

QHL#	Cluster	Fluorescens IC50 (µM)	Fluorescens pIC50	Absorbance pIC50	Fluorescence + Zn pIC50	TSA Δ °C	Mw (g/mol)	HBA	HBD	TPSA (Å^2)	LogD	LipE	QED
1	NA	0.32	6.50	6.06	6.5	6.77	346.40	6.00	2.00	95.09	1.65	4.85	0.65
2	26	0.35	6.45	6.10	6.47	3.7	434.91	7.00	2.00	104.32	1.56	4.89	0.52
3	26	0.38	6.42	6.15	6.41	5.22	382.47	7.00	2.00	108.22	2.34	4.08	0.54
4	NA	0.71	6.15	5.70	6.17	4	391.48	6.00	2.00	101.08	3.64	2.51	0.48
5	26	1.20	5.92	5.71	5.95	4.29	390.86	6.00	2.00	95.09	2.13	3.79	0.59
6	26	1.26	5.90	5.24	5.91	3.55	392.87	6.00	2.00	95.09	2.33	3.57	0.57
7	NA	1.29	5.89	<4.70	5.86	0.3	362.80	4.00	1.00	33.09	3.79	2.10	0.72
8	26	1.32	5.88	5.45	5.89	4.61	448.94	7.00	2.00	104.32	1.94	3.94	0.51
9	1	1.51	5.82	<4.70	5.8	0.15	344.39	7.00	1.00	112.49	2.84	2.98	0.79
10	15	1.58	5.80	4.80	5.78	0.3	493.51	9.00	1.00	100.65	3.27	2.53	0.46
11	4	1.66	5.78	5.41	5.77	1.92	400.94	5.00	2.00	64.15	3.21	2.57	0.83
12	NA	1.91	5.72	4.86	5.71	-0.15	287.75	5.00	1.00	55.63	4.04	1.68	0.80
13	NA	2.14	5.67	4.79	5.7	-1.91	392.50	7.00	0.00	59.31	3.28	2.39	0.61
14	1	2.24	5.65	<4.70	5.63	0.15	367.46	5.00	1.00	86.46	3.23	2.42	0.75
15	10	2.40	5.62	<4.70	5.59	-0.89	363.24	5.00	0.00	39.15	2.08	3.54	0.83
16	16	2.63	5.58	5.42	5.55	2.07	368.49	6.00	2.00	95.09	2.44	3.14	0.57
17	NA	2.63	5.58	<4.70	5.49	0.3	419.50	7.00	1.00	107.82	3.39	2.19	0.70
18	NA	2.82	5.55	5.25	5.57	1.92	485.49	10.00	1.00	100.23	2.53	3.02	0.56
19	1	2.82	5.55	4.79	5.53	0.3	355.46	6.00	2.00	105.91	3.72	1.83	0.75
20	16	2.82	5.55	5.24	5.5	4.15	404.93	6.00	2.00	123.33	2.84	2.71	0.54
21	25	2.95	5.53	5.25	5.55	2.66	330.36	6.00	2.00	78.35	2.96	2.57	0.67
22	15	3.09	5.51	<4.70	5.52	0.15	464.52	8.00	1.00	95.08	3.17	2.34	0.49
23	NA	3.09	5.51	<4.70	5.49	0.44	250.26	5.00	0.00	56.74	2.90	2.61	0.55
24	NA	3.09	5.51	5.52	5.26	4	387.47	7.00	2.00	98.33	1.94	3.57	0.55
25	1	3.16	5.50	<4.70	5.46	0	367.42	6.00	1.00	66.81	3.20	2.30	0.77
26	NA	3.24	5.49	5.11	5.45	4.44	428.52	7.00	2.00	104.32	2.58	2.91	0.51
27	NA	3.24	5.49	4.97	5.29	1.48	325.40	5.00	0.00	59.23	3.35	2.14	0.87
28	NA	3.24	5.49	4.81	5.25	0.15	330.42	4.00	1.00	96.50	3.95	1.54	0.73
29	25	3.31	5.48	5.53	5.49	1.92	360.84	6.00	2.00	78.35	3.88	1.60	0.64
30	15	3.47	5.46	<4.7	5.41	0.3	464.52	8.00	1.00	95.08	3.03	2.43	0.49
31	NA	3.47	5.46	<4.70	5.38	0	278.68	2.00	0.00	17.82	4.41	1.05	0.64
32	NA	3.47	5.46	<4.70	5.21	0.15	290.36	6.00	1.00	59.51	2.36	3.10	0.88
33	NA	3.72	5.43	5.510	5.42	0.74	376.41	7.00	1.00	80.49	1.61	3.82	0.75
34	19	3.89	5.41	<4.70	5.39	0.3	303.26	5.00	3.00	81.14	1.53	3.88	0.78
35	NA	3.98	5.40	4.85	5.34	0	466.59	6.00	0.00	77.11	4.25	1.15	0.49
36	NA	3.98	5.40	5.80	5.31	0.3	424.54	5.00	1.00	49.64	5.11	0.29	0.50
37	NA	4.37	5.36	<4.70	5.36	0.15	426.57	5.00	1.00	74.86	3.62	1.74	0.79
38	4	4.68	5.33	4.96	5.34	2.22	401.93	6.00	2.00	77.04	2.27	3.06	0.78
39	NA	4.68	5.33	5.12	5.33	0	443.97	6.00	0.00	62.74	4.67	0.66	0.58
40	10	4.68	5.33	<4.70	5.28	0.3	375.78	7.00	1.00	68.25	0.95	4.38	0.85
41	NA	4.68	5.33	5.81	5.11	0.44	421.49	6.00	0.00	85.71	4.74	0.59	0.56
42	NA	4.79	5.32	5.40	5.32	2.96	370.45	5.00	4.00	86.93	4.03	1.29	0.54
43	NA	5.89	5.23	5.12	5.27	3.26	387.47	7.00	1.00	89.54	2.07	3.16	0.44
44	NA	6.46	5.19	<4.70	5.18	0.44	288.41	4.00	0.00	60.50	3.69	1.50	0.85
45	NA	7.76	5.11	6.09	5.12	0.89	426.49	8.00	2.00	118.49	2.37	2.74	0.69
46	NA	7.76	5.11	5.82	5.11	0.3	474.59	7.00	3.00	94.03	3.51	1.60	0.54
47	NA	10.23	4.99	5.51	4.99	0.3	362.45	7.00	3.00	105.93	1.42	3.57	0.77
48	NA	17.38	4.76	5.47	4.77	0.3	409.54	5.00	0.00	78.81	2.94	1.82	0.78
49	19	17.78	4.75	5.65	4.74	0.44	251.33	3.00	2.00	43.84	3.99	0.76	0.75
50	NA	18.20	4.74	5.36	4.7	0.44	328.44	6.00	0.00	76.55	2.68	2.06	0.51

TSA: thermal shift assay; HBA, HBD: number hydrogen bond accepting and donating groups; TPSA , topological polar surface area; LipE: calculated from ($pIC_{50Fluorescence}$ – LogD); QED[4]: quantitative estimate of drug-likeness.

Table S2. Compound ELF screening history.

QHL#	Targets active	Targets Screened	Targets Active %	Enzyme Active	Enzymes Screened	Enzyme Active %
1	7	148	4.7	2	94	2.1
2	5	149	3.4	4	94	4.3
3	4	150	2.7	3	94	3.2
4	5	149	3.4	3	93	3.2
5	4	148	2.7	3	92	3.3
6	5	148	3.4	3	93	3.2
7	9	149	6.0	8	93	8.6
8	6	149	4.0	4	94	4.3
9	4	76	5.3	2	41	4.9
10	2	77	2.6	2	42	4.8
11	5	147	3.4	4	95	4.2
12	3	141	2.1	3	84	3.6
13	19	144	13.2	12	85	14.1
14	5	76	6.6	3	41	7.3
15	10	141	7.1	6	85	7.1
16	4	143	2.8	4	79	5.1
17	5	151	3.3	4	95	4.2
18	5	85	5.9	3	50	6.0
19	6	76	7.9	4	42	9.5
20	3	103	2.9	1	54	1.9
21	9	144	6.3	6	87	6.9
22	3	151	2.0	3	95	3.2
23	2	83	2.4	1	48	2.1
24	2	57	3.5	2	37	5.4
25	2	77	2.6	2	42	4.8
26	1	145	0.7	1	85	1.2
27	5	146	3.4	4	80	5.0
28	6	150	4.0	4	94	4.3
29	14	137	10.2	10	85	11.8
30	16	142	11.3	8	84	9.5
31	8	151	5.3	5	95	5.3
32	2	76	2.6	1	42	2.4
33	5	145	3.4	4	93	4.3
34	2	139	1.4	1	75	1.3
35	8	145	5.5	6	88	6.8
36	6	104	5.8	4	56	7.1
37	7	75	9.3	5	41	12.2
38	9	138	6.5	5	79	6.3
39	7	143	4.9	6	85	7.1
40	6	143	4.2	4	94	4.3
41	8	146	5.5	6	79	7.6
42	12	148	8.1	6	82	7.3
43	5	143	3.5	3	85	3.5
44	6	150	4.0	4	94	4.3
45	11	140	7.9	5	84	6.0
46	3	40	7.5	3	27	11.1
47	6	142	4.2	4	86	4.7
48	2	40	5.0	2	27	7.4
49	9	145	6.2	4	81	4.9
50	11	144	7.6	7	86	8.1

1. Nikolaou, A. *et al.* [3H]IVDE77, a novel radioligand with high affinity and selectivity for the insulin-regulated aminopeptidase. *Eur. J. Pharmacol.* **702**, 93–102 (2013).
2. Andersson, H. *et al.* Ligands to the (IRAP)/AT4 receptor encompassing a 4-hydroxydiphenylmethane scaffold replacing Tyr2. *Bioorg. Med. Chem.* **16**, 6924–6935 (2008).
3. Borhade, S. R. *et al.* Inhibition of insulin-regulated aminopeptidase (IRAP) by arylsulfonamides. *ChemistryOpen* **3**, 256–263 (2014).
4. Bickerton, G. R., Paolini, G. V., Besnard, J., Muresan, S. & Hopkins, A. L. Quantifying the chemical beauty of drugs. *Nat. Chem. 2011 42* **4**, 90–98 (2012).
